# *Toxoplasma gondii* associated with psychotic symptom load and cortisol in severe mental illness

**DOI:** 10.1038/s41537-025-00630-0

**Published:** 2025-05-26

**Authors:** Dimitrios Andreou, Nils Eiel Steen, Kjetil Nordbø Jørgensen, Stener Nerland, Thor Ueland, Laura A. Wortinger, Ina Drabløs, Tereza Calkova, Monica B. E. G. Ormerod, Linn Sofie Sæther, Ole A. Andreassen, Robert H. Yolken, Ingrid Agartz

**Affiliations:** 1 Division of Mental Health and Substance Abuse, Diakonhjemmet Hospital, Oslo, Norway; 2 Institute of Clinical Medicine, University of Oslo, Oslo, Norway; 3 Centre for Psychiatry Research, Department of Clinical Neuroscience, Karolinska Institutet & Stockholm Health Care Services, Stockholm Region, Stockholm, Sweden; 4 Division of Mental Health and Addiction, Oslo University Hospital, Oslo, Norway; 5 Division of Mental Health and Addiction, Vestre Viken Hospital Trust, Drammen, Norway; 6 Research Institute of Internal Medicine, Oslo University Hospital, Rikshospitalet, Oslo, Norway; 7 Thrombosis Research Center (TREC), Division of internal medicine, University hospital of North Norway, Tromsø, Norway; 8 Department of Psychology, Oslo New University College, Oslo, Norway; 9 Region Vastmanland – Uppsala University, Centre for Clinical Research, Vastmanland Hospital Vasteras, Västerås, Sweden; 10 Centre for Precision Psychiatry, Division of Mental Health and Addiction, Oslo University Hospital and University of Oslo, Oslo, Norway; 11 Stanley Division of Developmental Neurovirology, Department of Pediatrics, Johns Hopkins University School of Medicine, Baltimore, MD USA

**Keywords:** Schizophrenia, Biomarkers

## Abstract

*Toxoplasma gondii* (TG) is a prevalent parasite that establishes lifelong latency after primary infection. TG has been linked to severe mental illness (SMI), potentially through dopamine dysregulation in the brain. There is a bidirectional interaction between dopamine and the hypothalamic-pituitary-adrenal axis, where dopamine may influence cortisol regulation and cortisol may affect dopamine release. We hypothesised that TG would be associated with elevated circulatory cortisol levels, increased severity of psychotic symptoms, and structural brain aberrations in SMI. Our study included 765 patients with SMI (515 with schizophrenia spectrum disorders and 250 with bipolar disorders) and 541 healthy controls (HC). TG immunoglobulin G seropositivity and circulatory cortisol concentrations were measured with immunoassays, and T1-weighted MRI scans were processed using FreeSurfer. Psychotic symptom scores were evaluated using the Positive and Negative Syndrome Scale. In SMI, TG seropositivity was associated with higher cortisol levels (*p* = 0.002), but not in HC. Seropositive patients had lower total psychotic symptom scores (*p* = 0.006) than seronegative patients, driven by the schizophrenia subgroup (*p* = 0.002). This effect was observed for positive, negative, and general psychotic symptom scores, but only for patients with an illness duration of 10 years or more. In an exploratory analysis, TG seropositivity was nominally associated with smaller thalamus, nucleus accumbens, and middle temporal volumes in SMI, and with smaller fusiform, parahippocampal, and pars triangularis volumes in HC. In conclusion, TG exposure in SMI was linked to elevated cortisol levels and reduced psychotic symptom scores, suggesting that its impact on SMI may be more complex and context-dependent than previously assumed.

## Introduction

*Toxoplasma gondii* (TG) is a highly prevalent intracellular parasite, with seroprevalence rates varying between 30% to 65% across human populations. Infection in humans occurs primarily through the ingestion of oocysts from environments contaminated by cat feces, or by consuming undercooked meat containing tissue cysts^1^. In immunocompetent hosts, the primary infection is usually asymptomatic or produces mild flu-like illness; however, for those with a weakened immune system or congenital infection, TG can lead to serious health issues, including central nervous system involvement^2,3^. Once contracted, the parasite typically establishes a lifelong latency by forming persistent cysts in tissues such as the brain, eyes, heart, and muscles^1^.

A substantial body of research has investigated associations between TG infection and psychiatric disorders, with a primary focus on schizophrenia (SZ) and bipolar disorder (BD), reflecting significant scientific interest in the potential impact of this parasite on mental health. Given the extensive number of original studies, we here aim to mainly summarize key meta-analytic findings. Notably, Sutterland et al.^4^ reported an almost twofold increase in the odds of SZ among individuals with TG infection, a finding subsequently replicated in a more recent meta-analysis^5^. Further supporting this association, a large-scale study using UK Biobank data demonstrated that TG seropositivity at baseline was predictive of SZ incidence at a 12-year follow-up^6^. The association between TG and BD has also been confirmed in multiple meta-analyses, though with somewhat smaller effect sizes than those observed for SZ^4,7,8^. Importantly, the implications of TG infection in psychiatric disorders are not limited to severe mental illness (SMI); meta-analytic evidence has also demonstrated associations with obsessive-compulsive disorder and addiction^4^. Additionally, prior research suggests potential links between TG and other psychiatric conditions, including anxiety disorders, personality disorders, primarily antisocial and borderline, and PTSD. These findings, as reviewed by Flegr^9^, underscore a broader role for TG in psychiatric disorders beyond SMI.

These observed associations between TG and psychiatric disorders may be attributable to the parasite’s ability to infect the brain and alter the functioning of multiple neurotransmitter systems, including dopamine, which causes changes in dopaminergic signalling and ultimately changes in behaviour^10^. In particular, TG has been shown to increase dopamine levels in the brain by upregulating the production of tyrosine hydroxylase, a key enzyme in dopamine synthesis^10^. This parasitic modulation of the dopamine system could contribute to the association between TG and SMI^4,11,12^. The dopamine hypothesis continues to be one of the most influential frameworks for understanding SMI, especially SZ. Extensive research points to dopamine as a key factor underlying mechanisms and therapeutic interventions in SZ^13–15^, but also BD has been linked to a dysregulation of the dopamine system with elevated dopamine activity shown during manic episodes, and dopamine receptor gene variations increasing BD susceptibility^16,17^.

Notably, dopamine dysregulation has been identified as a transdiagnostic mechanism underlying shared symptoms in SZ and BD, particularly psychotic symptoms and reward processing deficits. Jauhar et al.^18^ demonstrated that both SZ and psychotic BD are characterized by elevated dopamine synthesis capacity in the striatum, with this increase significantly correlating with the severity of psychotic symptoms, highlighting dopamine dysregulation as a potential contributor to shared psychotic features. Additionally, corticostriatal functional dysconnectivity has been observed in both SZ and psychotic BD, further supporting a common neurobiological substrate across psychotic disorders^19^. Moreover, aberrant reward processing has been consistently reported in both SZ and BD, implicating dysfunction in reward-related dopamine signaling as a shared pathophysiological feature of these disorders^20^. It should, however, be emphasized that numerous risk factors have been associated with an increased striatal dopaminergic function in SMI, mainly SZ, including genetic variations, obstetric complications, stress, and trauma^15^. For instance, Mizrahi et al. reported that stress induces heightened dopamine release in the associative and sensorimotor striatum in patients at clinical high risk for psychosis as well as in those with SZ, suggesting a sensitized dopaminergic response to psychological stress^21^. Further, it has been shown that there is a bidirectional association between inflammatory processes, another major framework for understanding SMI, and dopamine dysfunction, and in this context, it has been suggested that neuroinflammation is linked to an overstimulation of dopaminergic as well as glutamatergic neurons^22^.

Dopaminergic dysfunction can also dysregulate cortisol, which appears to be altered in psychotic disorders. Cortisol is a steroid hormone produced in the adrenal cortex. Its secretion is regulated by the hypothalamic-pituitary-adrenal (HPA) axis. In this process, the hypothalamus releases corticotropin-releasing hormone (CRH), which stimulates the anterior pituitary gland to secrete adrenocorticotropic hormone (ACTH). ACTH, in turn, acts on the adrenal cortex to promote cortisol release. Often referred to as the body’s stress hormone, cortisol plays a crucial role in managing stress responses, regulating immune functions, and maintaining glucose and protein homeostasis^23^. Cortisol levels typically peak 30–40 minutes after awakening, a phenomenon known as the cortisol awakening response (CAR), and then gradually decrease throughout the day; of note, both baseline cortisol levels and the CAR are reported to be dysregulated in individuals with SMI. Comprehensive meta-analyses revealed elevated morning cortisol concentrations in SZ, first-episode psychosis, and BD^24,25^ as well as a flattened CAR in patients with SZ and first-episode psychosis^26^. Notably, cortisol levels rise not only upon awakening but also in response to stress; however, this stress-induced response has been observed to be attenuated in individuals with SMI, particularly those with SZ^27^. The neural-diathesis-stress model suggests that individuals with a predisposition (diathesis) may develop SZ when exposed to significant stress, partly due to HPA axis dysregulation interacting with other neurobiological systems and contributing to its pathogenesis^27^. Interestingly, there is bidirectional interplay between the HPA axis and the dopaminergic system. For instance, human studies have shown that acute stress and subsequent cortisol release are linked to enhanced dopamine transmission, particularly in the mesolimbic pathway^28,29^, and that treatment with corticotropin-releasing hormone enhances dopamine release^30^. Further, previous animal^31^ and human studies^32,33^ with relatively small sample sizes have investigated the putative associations between TG and cortisol levels, with somewhat conflicting results. TG may contribute to the HPA axis dysregulation in SMI, possibly through the parasite’s well-documented dopaminergic effects^10^.

TG-related neurochemical alterations, such as cortisol dysregulation, may increase the clinical burden in SMI and potentially contribute to structural brain changes. A recent meta-analysis showed that latent toxoplasmosis is linked to increased severity of psychotic symptoms in SZ patients^11^, a clinically important finding given that symptom severity is a major predictor of functional outcome, quality of life, and long-term prognosis in this population. Additionally, an MRI study found TG-related reductions of cortical and subcortical grey matter volumes among SZ patients^34^. However, findings remain inconsistent, particularly regarding TG’s impact on brain structure and its differential effects between SMI and healthy controls (HC).

The present study aims to explore the potential role of latent TG infection in the pathophysiology of SMI. Specifically, we sought to examine associations between TG seropositivity and (i) circulating cortisol concentrations, (ii) severity of psychotic symptoms, and (iii) structural brain measures assessed by MRI, in individuals with SMI and in HC. We hypothesized that in SMI, latent toxoplasmosis, indicated by TG immunoglobulin G (IgG) seropositivity, would be associated with i) increased circulatory cortisol levels, ii) greater severity of psychotic symptoms, and (iii) smaller grey matter volumes in the brain. We further hypothesized an absence of such associations in HC. The hypothesized vulnerability in SMI may be attributed to immunological dysfunction^35–37^ and increased blood-brain barrier permeability^38,39^ in both SZ and BD, that could heighten susceptibility to environmental hazards, including parasites.

## Patients and methods

### Participants

Participants for this study were selected from the Thematically Organized Psychosis (TOP) research study in the period 2003–2017. Patients, either outpatients or inpatients, were recruited from psychiatric clinics located in Oslo, Norway. HC were randomly sampled from the same geographical region using the Norwegian National Population Register. Patients underwent diagnostic assessments using the Structured Clinical Interview (SCID-I) for the Diagnostic and Statistical Manual of Mental Disorders, fourth edition (DSM-IV)^40^. Only those diagnosed with SZ or BD spectrum disorders were included. For the HC group, participants were screened for SMI with an interview, including the Primary Care Evaluation of Mental Disorders (Prime-MD)^41^, and those with current or past psychiatric disorders, including substance use disorder (including alcohol use disorder) or those with first-degree relatives diagnosed with SMI, were excluded. Additionally, any participants with a history of moderate or severe head injury, neurological disorders, or medical conditions potentially impacting brain function were excluded.

The TG IgG-clinical data analysis encompassed a sample size of 1306 participants including 765 patients with SMI, i.e., 515 patients with SZ spectrum disorders (SZ, *n* = 295; schizophreniform disorder, *n* = 31; schizoaffective disorder, *n* = 74; delusional disorder, *n* = 34; brief psychotic disorder, *n* = 5; psychotic disorder not otherwise specified, *n* = 76), and 250 patients with BD spectrum disorders (BD I, *n* = 169; BD II, *n* = 69; BD not otherwise specified, *n* = 12), and 541 HC. For the TG IgG-cortisol analysis, the sample included 660 patients with SMI, i.e., 426 patients with SZ spectrum disorders and 234 patients with BD spectrum disorders, and 193 HC, while the TG IgG-MRI analysis involved 414 patients with SMI, i.e., 250 patients with SZ spectrum disorders and 164 patients with BD spectrum disorders, and 404 HC.

The authors assert that all procedures contributing to this work comply with the ethical standards of the national and institutional committee on human research and with the Helsinki Declaration. The study was approved by the Regional Committee for Medical Research Ethics, South East Norway, and the Norwegian Data Protection Authority. All participants gave written informed consent.

### Assessment of demographic, clinical, and medication use measures

In this study, we used years of education as a proxy measure for socioeconomic status for both patients and HC. Education level serves as a socioeconomic status indicator, capturing the transition from parental to individual socioeconomic status^42^. All participants were evaluated for alcohol use through the Alcohol Use Disorder Identification Test (AUDIT)^43^ and drug use through the Drug Use Disorder Identification Test (DUDIT)^44^. Additionally, we assessed full-scale current intelligence quotient (IQ) using a licensed, translated version of the Wechsler Abbreviated Scale of Intelligence (WASI)^45^. Patients were further evaluated using the Positive and Negative Syndrome Scale (PANSS)^46^, the Young Mania Rating Scale (YMRS)^47^, and the Inventory of Depressive Symptoms, clinician-rated (IDS-C)^48^. The duration of illness (DOI) was defined as the period from the first psychotic episode for SZ spectrum patients and the first affective episode for BD spectrum patients. Finally, information on the current use of antipsychotics, antiepileptics, antidepressants, and lithium was gathered through clinical interviews and hospital records. For patients on antipsychotics, we calculated the current chlorpromazine equivalent doses (CPZ) in mg/day^49,50^.

### TG and cortisol measurements

Serology measurements were obtained by standard procedures at the Stanley Neurovirology Laboratory (Johns Hopkins University School of Medicine, Baltimore, MD, USA). TG IgG antibody concentrations were measured by solid-phase immunoassays and expressed as dichotomous, seropositive/seronegative (TG+/TG–) measures. The amount of antibody was expressed in terms of the ratio of optical density of the test sample to that of the standard sample, and the cut-offs for seronegativity/seropositivity were based on standards run with each sample^51^. Serum cortisol level was measured using a competitive luminescence immunoassay (Immulite 2000xpi, Siemens Healthineers, Erlangen, Germany) at the Hormone Laboratory, Department of Medical Biochemistry, Oslo University Hospital, Norway^52^.

### Brain MRI acquisition and analysis

T1-weighted MRI brain scans were acquired using two different scanners: a 1.5 T Siemens MAGNETOM Sonata scanner with a standard head coil (278 patients and 143 HC) and a 3 T General Electric Signa HDxt scanner with an 8-channel head coil (136 patients and 261 HC). For the 1.5 T Siemens MAGNETOM Sonata, two T1-weighted scans were obtained and averaged to enhance the signal-to-noise ratio (SNR). Details of the T1-weighted MRI sequences are provided in Suppl. Table 1.

The MRI data were processed using FreeSurfer v6.0.0^53^. As part of an exploratory analysis, we examined the volumes of the caudate, putamen, nucleus accumbens, pallidum, thalamus, amygdala, and hippocampus, along with regional cortical volumes defined by the Desikan–Killiany atlas^54^. All T1-weighted images were inspected by trained research assistants and excluded if major artifacts, e.g., due to excessive movement, were present. Surface reconstructions were manually edited in the event of reconstruction errors following standard FreeSurfer procedures. Voxel-wise segmentations were routinely inspected to rule out segmentation errors^55^.

### Statistics

#### Clinical sample

In the bivariate analysis of HC (*n* = 541), we used chi-square tests for categorical variables and t-tests for quantitative variables to examine group differences between TG+ and TG– HC in terms of sex, age, education years, AUDIT score, DUDIT score, and IQ. Further, for patients with SMI (*n* = 765), we also assessed group differences in DOI, PANSS total score, YMRS, IDS-C, and medication variables using the same statistical methods (Table 1). Next, in our multivariable models (analyses of covariance; ANCOVAs) among patients, we explored the putative associations between TG status (TG+/TG–) and PANSS total, positive, negative, and general scores, and YMRS score, whilst controlling for sex and age. Finally, we repeated the analyses separately for SZ and BD spectrum disorders, and in line with a recent meta-analysis^11^, separately for SZ patients with short (<10 years) and long (from 10 years) DOI.

**Table 1:** Group differences between *Toxoplasma gondii* seropositive and seronegative participants.

	TG+		TG–		*P* value^b^	Correlation with cortisol	
	N^a^	Mean (SD) or %	N^a^	Mean (SD) or %		Direction (+ or –)	*P* value^c^
Patients with SMI							
Sex (% women)	159	42.8	606	48.8	0.172	+^d^	**0.001**
Age (years)	159	32.2 (11.7)	606	32 (10.5)	0.850	–	**0.002**
Education years	138	12.7 (2.3)	556	12.6 (2.6)	0.640	+	0.953
DOI (years)	156	8.7 (7.7)	586	9.4 (9.1)	0.308	–	0.092
PANSS total score	156	54 (14.5)	594	57.7 (17.3)	**0.012**	–	0.820
YMRS score	127	3.7 (4)	527	4.9 (5.3)	**0.004**	+	0.201
IDS-C	108	16.6 (11.1)	454	18.8 (12.3)	0.099	+	0.875
IQ	112	101.5 (14.3)	490	101.4 (15)	0.939	+	**0.015**
On antipsychotics (%)	159	74.2	606	74.3	0.991	+	0.978
On antidepressants (%)	159	32.7	606	32	0.868	–	0.125
On antiepileptics (%)	159	21.4	606	21	0.907	+	0.107
On lithium (%)	159	10.7		8.1	0.298	+	0.388
CPZ (mg/day)	115	320.6 (226.2)	446	314.7 (253.6)	0.821	–	0.159
AUDIT score	116	7 (6.3)	437	7.5 (6.9)	0.436	+	0.066
DUDIT score	124	3.3 (6.8)	466	3.4 (7.2)	0.872	+	0.252
HC							
Sex (% women)	126	42.9	415	44.6	0.733	+^d^	**0.030**
Age (years)	126	33.3 (8.8)	415	33.4 (9.2)	0.916	–	**0.007**
Education years	126	14.2 (2.1)	415	14.4 (2.2)	0.277	–	0.060
IQ	126	112.9 (10.8)	409	113.5 (10.1)	0.613	–	0.098
AUDIT score	92	5.5 (3.1)	221	5.6 (3.2)	0.713	+	0.327
DUDIT score	95	0.2 (0.6)	223	0.3 (1.5)	0.483	–	0.361

Group differences between *Toxoplasma gondii* (TG) immunoglobulin G (IgG) seropositive (TG+) and seronegative (TG–) patients with severe mental illness (SMI) in sex, age, education years, duration of illness (DOI), Positive and Negative Syndrome Scale (PANSS) total score, Young Mania Rating Scale (YMRS) score, Inventory of Depressive Symptoms, clinician rated, score (IDS-C), intelligent quotient (IQ), the percentage of patients on antipsychotics, antidepressants, antiepileptics and lithium as well as the chlorpromazine equivalent doses (CPZ) among patients on antipsychotics, alcohol use disorder identification test (AUDIT) and drug use disorder identification test (DUDIT) scores. Group differences between TG+ and TG– healthy controls (HC) in sex, age, education years, IQ, AUDIT, and DUDIT scores. In addition, utilizing a subsample of 660 SMI patients (136 TG+) and 193 HC (72 TG+), correlation analysis was conducted between all individual variables and cortisol concentrations. *P* values < 0.05 are shown in bold.

^a^Number of participants with data in each variable.

^b^Chi-square test or *t* test (Welch’s *t* test in case of unequal variance).

^c^Point-biserial correlations for binary variables; Spearman’s correlations for quantitative variables.

^d^Women had significantly higher cortisol concentrations than men.

#### Cortisol sample

**Fig. 1:**
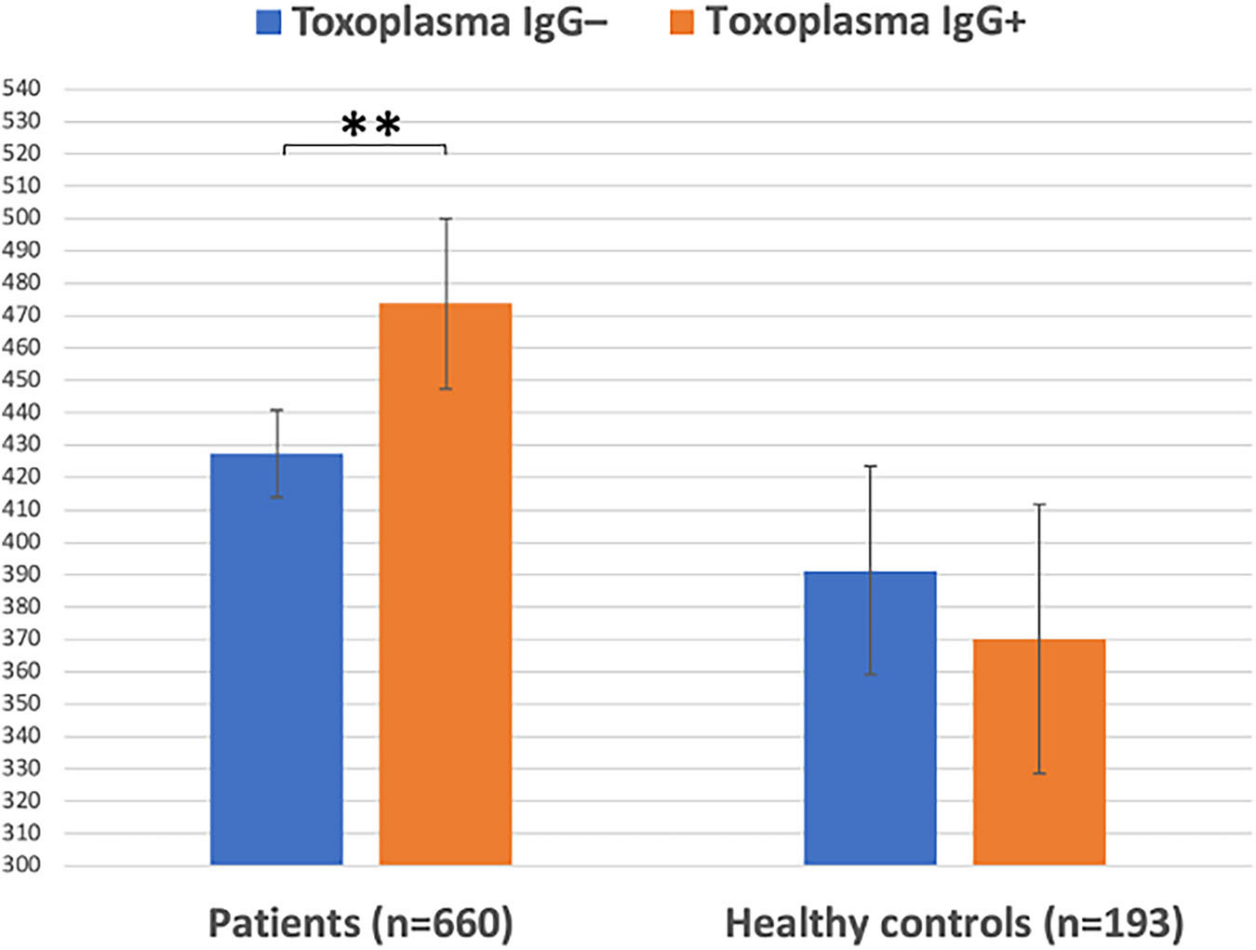
*Toxoplasma gondii* and cortisol concentrations. Circulatory cortisol concentrations in nmol/L in *Toxoplasma gondii* immunoglobulin G seronegative (IgG–) and seropositive (IgG+) patients with severe mental illness (left), and IgG– and IgG+ healthy controls (right). Adjusted means from age- and sex-adjusted analyses of covariance are shown. Error bars represent the 95% confidence intervals calculated using standard errors. **p < 0.005.

In the smaller cortisol sample, we applied ANCOVAs separately in 660 SMI patients and 193 HC to examine the main effects of TG status (TG+/TG–) on cortisol concentrations. Analyses were controlled for variables that significantly correlated with cortisol concentrations (Table 1 and Fig. 1). Finally, we explored the putative correlations between selected immune markers and cortisol concentrations (Suppl. notes).

#### MRI sample

For the MRI sample, comprising 414 SMI patients and 404 HC, and for patients and HC separately, we performed sex-, age-, scanner- and estimated intracranial volume (ICV)-adjusted ANCOVAs on the following bilateral subcortical structures: caudate, putamen, nucleus accumbens, pallidum, thalamus, amygdala, and hippocampus, applying a false discovery rate (FDR) of 5% by hemisphere to correct for multiple testing^56^. Further, we extracted cortical grey matter volumes from 34 bilateral regions based on the Desikan-Killiany FreeSurfer atlas^54^, which were used as dependent variables in 34 sex-, age-, and scanner-adjusted ANCOVAs per hemisphere, and applied a 5% FDR by hemisphere to correct for multiple testing^56^.

#### Long vs. short DOI analysis

Finally, we performed two separate analyses of variance (ANOVAs) to examine differences in PANSS total scores and cortisol concentrations between TG + SZ patients currently on antipsychotics with long and short DOI.

All tests were two-sided. ANCOVA assumptions for main models with statistically significant results are shown in suppl. notes. We conducted all the analyses with IBM SPSS Statistics 28.

## Results

### Clinical sample

In the bivariate analysis, TG + SMI patients did not significantly differ from TG– SMI patients in sex distribution, age, education years, DOI, IDS-C score, IQ, current medication use, CPZ, AUDIT, or DUDIT scores. TG + SMI patients had lower PANSS total score (*p* = 0.012) and YMRS score (*p* = 0.004) compared with TG– SMI patients. TG+ and TG– HC did not significantly differ in sex distribution, age, education years, IQ, AUDIT, or DUDIT scores (Table 1).

In our multivariable models (ANCOVAs) following the significant bivariate association between TG status and PANSS total score, TG + SMI patients had lower PANSS total score than TG– SMI patients, whilst adjusting for age and sex, F(1,746) = 7.555, *p* = 0.006, partial eta squared (*η*^2^) = 0.010. Stratifying by diagnosis, there was still a significant association of TG status with PANSS total score in SZ, with lower PANSS total score in TG+ than in TG– patients, F(1497) = 9.830, *p* = 0.002, *η*^2^ = 0.019, but not in BD, F(1,245) = 0.243, *p* = 0.622, *η*^2^ = 0.001. In SZ, the association remained significant after further adjustment for current antipsychotic use or CPZ (*p* = 0.002 for both).

**Fig. 2:**
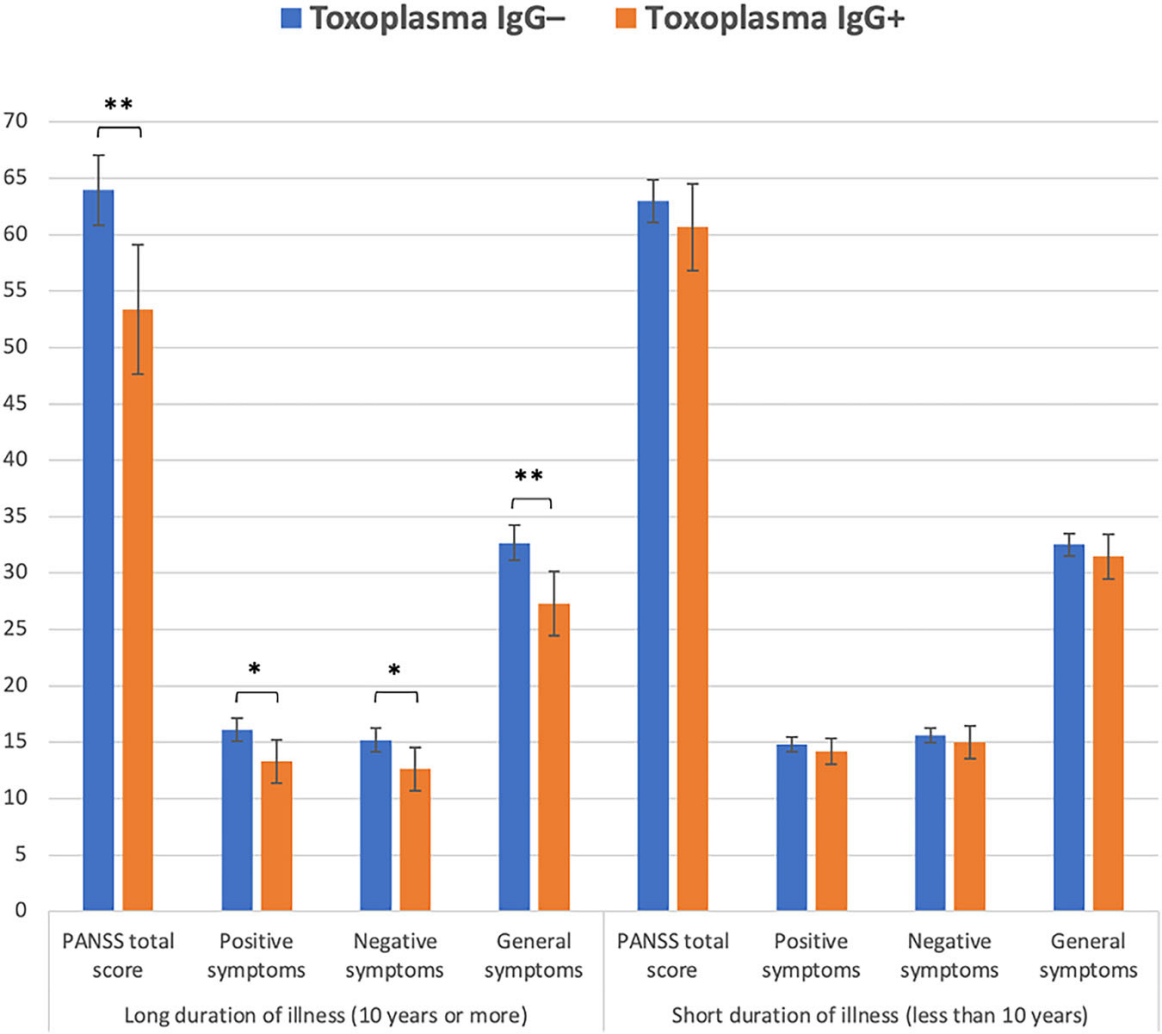
*Toxoplasma gondii* and psychotic symptom scores. Positive and Negative Syndrome Scale (PANSS) total, positive, negative, and general symptom scores in *Toxoplasma gondii* immunoglobulin G seronegative (IgG–) and seropositive (IgG+) patients with schizophrenia spectrum disorders with long (left) and short (right) duration of illness. 151 patients with long duration of illness (116 seronegative/35 seropositive) and 337 patients with short duration of illness (271 seronegative/66 seropositive) are included in the analysis. Adjusted means from age- and sex-adjusted analyses of covariance are shown. Error bars represent the 95% confidence intervals calculated using standard errors. *p < 0.05, **p < 0.005.

Next, in SZ, we followed up the significant TG-PANSS total score association, exploring the positive, negative, and general psychotic symptom scores. In sex- and age-adjusted ANCOVAs, TG status was associated with all three subscores, with TG+ patients showing lower scores than TG– patients (*p* = 0.012, 0.022, and 0.02, respectively). Finally, we studied separately SZ patients with short (<10 years, *n* = 337) and long (from 10 years, *n* = 151) DOI. Among SZ patients with long DOI, TG status was still associated with total, positive, negative, and general psychotic symptom scores, *p* = 0.002, 0.013, 0.021, and 0.001, respectively, while the corresponding *p* values for SZ patients with short DOI were 0.285, 0.327, 0.438, and 0.346, respectively (Fig. 2).

### Cortisol sample

As shown in Table 1, among both SMI patients and HC, age was inversely correlated with cortisol concentrations, assessed with Spearman’s correlations, *r*_s_ = −0.118, *p* = 0.002 and *r*_s_ = −0.194 *p* = 0.007 for SMI and HC, respectively. In addition, in both samples, women had higher cortisol concentrations than men, assessed with point-biserial correlations, *r*_pb_ = 0.124, *p* = 0.001 and *r*_pb_ = 0.156, *p* = 0.030 for SMI and HC, respectively. Further, among SMI patients, IQ was positively correlated with cortisol concentrations, *r*_s_ = 0.106, *p* = 0.015.

In our age- and sex-adjusted ANCOVA among SMI patients, TG IgG tatus was significantly associated with cortisol levels, F(1,656) = 9.455, *p* = 0.002, *η*^2^ = 0.014, with TG+ patients showing higher levels than TG– patients (Fig. 1). Controlling for IQ, the association remained significant, F(1,524) = 5.455, *p* = 0.020, *η*^2^ = 0.010. In our age- and sex-adjusted ANCOVA among HC, TG status was not associated with cortisol levels, F(1,189) = 0.620, *p* = 0.432, *η*^2^ = 0.003 (Fig. 1).

### MRI sample

#### Subcortical structures

**Fig. 3:**
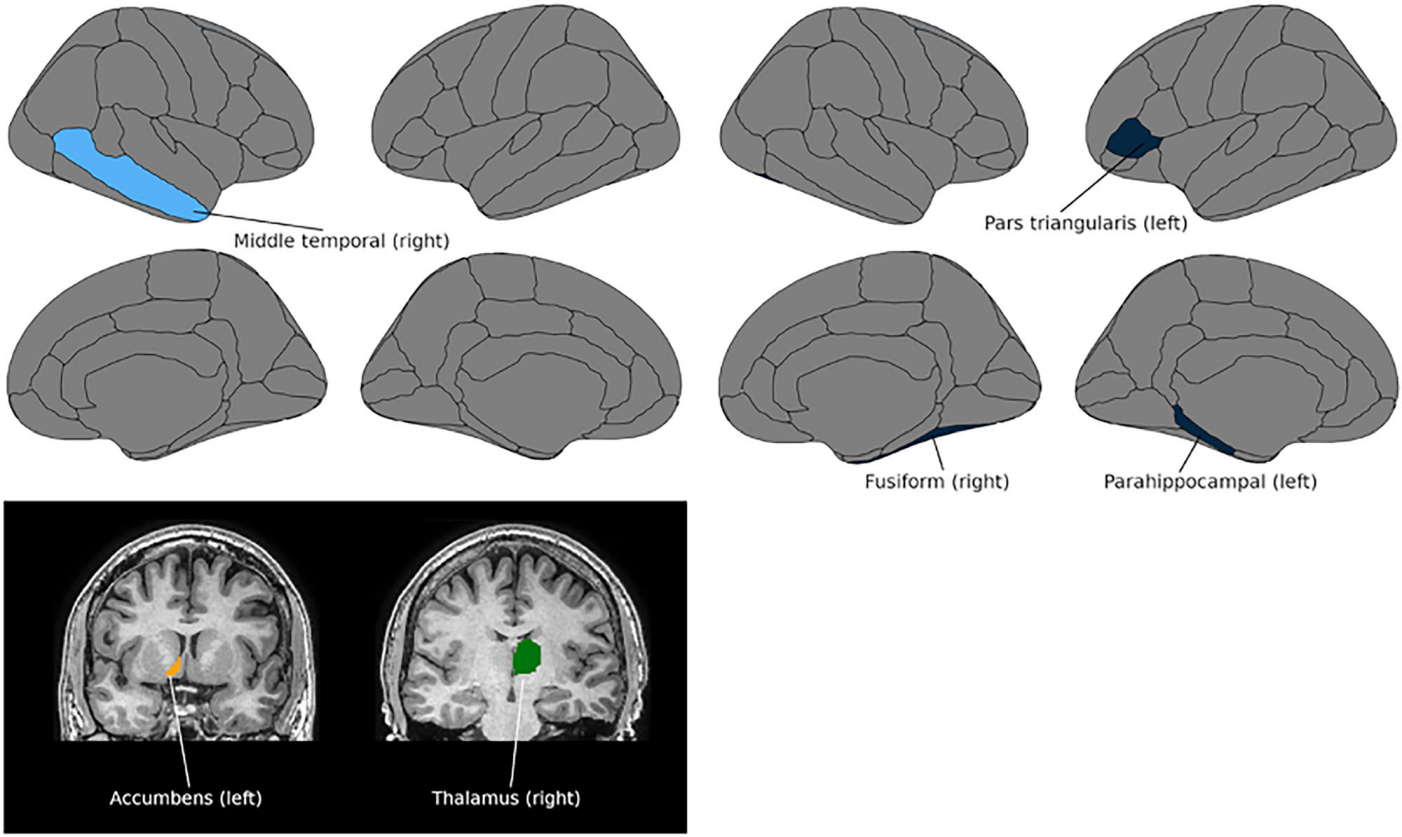
*Toxoplasma gondii* and brain structure. Exploratory analysis of *Toxoplasma gondii* and regional cortical and subcortical brain volumes. Left: Regional cortical (up) and subcortical (down) grey matter volumes that differed between *Toxoplasma gondii* immunoglobulin G seropositive (TG+) and seronegative (TG–) patients with severe mental illness. Right: Regional cortical volumes that differed between TG+ and TG– healthy controls. Seropositivity was nominally (*p* < 0.05) associated with smaller volumes for all illustrated regions.

We found nominally significant associations between TG seropositivity and right thalamus, F(1,408) = 5.258, *p* = 0.022, *η*^2^ = 0.013, and left nucleus accumbens, F(1,408) = 4.317, *p* = 0.038, *η*^2^ = 0.010, among SMI patients with smaller volumes in TG+ than TG– patients (Table 2 & Fig. 3). TG seropositivity was not associated with any subcortical volumes among HC. None of the nominally significant associations survived the FDR correction.

**Table 2:** *Toxoplasma gondii* and brain structure.

Cortical/subcortical volumes (mm^3^)	TG+	TG–	*P* value
SMI patients			
Thalamus (right)	6945	7080	0.022
Middle temporal (right)	12632	13072	0.030
Accumbens (left)	580	602	0.038
HC			
Fusiform (right)	10150	10493	0.013
Parahippocampal (left)	2219	2289	0.043
Pars triangularis (left)	4081	4230	0.049

The results of the analyses of covariance of Toxoplasma gondii status, seropositive (TG+) vs. seronegative (TG–) participants, on left and right subcortical and regional cortical volumes in patients with severe mental illness (SMI) and healthy controls (HC). Nominally significant associations (*p* < 0.05) are shown. No associations survived false discovery rate (FDR) correction for multiple testing.

#### Regional cortical volumes

We found nominally significant associations of TG seropositivity with the right middle temporal region, F(1,409) = 4.762, *p* = 0.030, *η*^2^ = 0.012, in SMI patients, and with the right fusiform, F(1,399) = 6.179, *p* = 0.013, *η*^2^ = 0.015, left parahippocampal, F(1,399) = 4.128, *p* = 0.043, *η*^2^ = 0.010, and left pars triangularis, F(1,399) = 3.910, *p* = 0.049, *η*^2^ = 0.010, regions among HC, with smaller volumes in TG+ than in TG– participants (Table 2 & Fig. 3). None of the nominally significant associations survived the FDR correction.

### Long vs. short DOI analysis

Further, among TG + SZ patients currently on antipsychotics, we ran an ANOVA to examine differences in PANSS total scores between those with a short (*n* = 54) and long (*n* = 27) DOI. Patients with a long DOI showed significantly lower total psychotic symptom scores than patients with short DOI, F(1,79) = 4.551, *p* = 0.036, *η*^2^ = 0.054. Finally, among TG + SZ patients currently on antipsychotics, we performed a separate ANOVA to assess potential differences in cortisol concentrations between TG + SZ patients with short (*n* = 55) and long (*n* = 26) DOI. There was no significant difference in cortisol concentrations between the two patient groups, F(1,79) = 0.907, *p* = 0.344, *η*^2^ = 0.011.

## Discussion

We found that TG seropositivity was associated with elevated cortisol levels in the SMI patient group, but not in HC, potentially reflecting increased vulnerability in patients to the effects of TG. Indeed, we have previously reported higher TG antibody levels in patients with SMI compared to HC, correlated with indices of enhanced systemic inflammation and central nervous system pathology^57^. Notably, we previously found no associations between TG antibody concentration levels and symptom severity^57^, consistent with the current study, showing no detrimental effect of TG on symptom scores but rather an association with lower symptom severity.

The potential association of TG with cortisol levels has been investigated in a limited number of human and animal studies with relatively small sample sizes, yielding conflicting results. In particular, TG seropositivity has been associated with elevated cortisol concentrations in both men and women^32^. However, this association was not observed in a study conducted on a wild mammalian species^31^. Interestingly, among TG+ individuals in the human study, TG antibody concentrations were positively correlated with cortisol concentrations^32^. A previous study of hospitalized patients with SZ reported no significant association between TG seropositivity and cortisol levels, but noted 18% higher cortisol concentrations in TG+ relative to TG– patients, which is consistent with our finding^33^. In view of the bidirectional relationship between dopamine and the HPA axis^58,59^, alongside the established effect of TG on increasing dopamine levels^10^, it is plausible that the observed TG-cortisol association is mediated via dopaminergic pathways.

In our SMI sample, TG+ patients exhibited lower psychotic symptom scores than TG– patients, driven by patients with SZ and related to long DOI. Although this finding was unexpected and not in line with our hypothesis, a recent meta-analysis showed that TG seropositivity was associated with more positive psychotic symptoms in the case of short duration of SZ only (<10 years)^11^. Interestingly, the majority of reports (6/8) studying patient groups with a longer DOI (>10 years) showed non-significantly lower positive symptom scores in association with TG seropositivity^11^. In our post-hoc analyses, stratifying by diagnosis, it became evident that among SZ patients, TG seropositivity was associated with lower psychotic symptom scores, interestingly both total, positive, negative and general symptom scores, while further stratification by DOI showed that these inverse associations were only present in SZ with a long DOI (10 years or more). We speculate that the inverse TG-psychotic symptom association stems from an augmented effect of antipsychotics in TG+ patients relative to TG– patients, which is more evident among patients who have a longer DOI, reflecting a longer exposure to antipsychotics. Indeed, certain psychotropic agents, mainly antipsychotics, exhibit anti-TG activity^60^, potentially reducing the pathogen’s impact on the brain and thereby decreasing psychotic symptoms in long-term patients. Notably, among TG + SZ patients receiving antipsychotics, those with a longer DOI exhibited significantly lower PANSS total scores compared to those with a shorter DOI, which may support the notion of an enhanced antipsychotic effect with prolonged DOI in TG + SZ patients.

To our knowledge, there are two previous reports investigating the putative associations between TG latent infection and brain structure in patients with SZ. Horacek et al. found that TG+ patients with SZ had smaller caudate, thalamus, cerebellum, median cingulate, parahippocampal and lingual gyrus, posterior cingulate, and fusiform gyrus compared to TG– patients, with no volumetric difference between TG+ and TG– HC^34^. The study sample was small and included 44 patients with SZ (12 TG + ) and 56 HC (13 TG + ). Further, Cobia et al. studied 77 SZ patients (5 TG + ) and 36 HC (11 TG + ), and reported no differences in cortical thickness between TG+ and TG– SZ patients or TG+ and TG– HC; interestingly, in SZ, TG IgG levels were associated with thinner cortex of the right superior parietal region^61^. Our study had a considerably larger sample size and identified nominally significant associations between TG seropositivity and smaller left nucleus accumbens, right thalamus, and right middle temporal cortical volume among SMI patients, and right fusiform, left parahippocampal, and left pars triangularis cortical volumes in HC. Interestingly, all subcortical structures studied showed (not statistically significant) smaller volumes in TG+ patients compared to TG– patients. Although none of these associations survived FDR correction and are therefore not elaborated upon here, our findings cannot exclude a potential link between TG exposure and structural brain alterations.

TG has been implicated in structural brain damage through mechanisms involving chronic neuroinflammation, dynamic cyst formation, and extracellular matrix disruption, as demonstrated by animal studies and our previous research in SMI. Baker et al.^62^ reported that pre-existing TG infections were associated with an exacerbated immune response and increased lesion size following traumatic brain injury, suggesting that TG infection may potentiate structural brain damage. Similarly, Schneider et al.^63^ demonstrated that TG infection induces monocyte recruitment to the brain, predominantly near sites of infection. Moreover, Meurer et al.^64^ found that TG infection disrupts perineuronal nets—specialized extracellular matrix structures that stabilize synaptic connections—across multiple cortical regions. Further, Watts et al. showed that TG bradyzoites within brain tissue cysts, historically considered dormant, exhibited unexpected biological activity, displaying both asynchronous and synchronous replication patterns^65^. This dynamic behavior of tissue cysts may contribute to the brain volume loss observed in TG-infected individuals. In our previous studies utilizing overlapping samples with the present cohort, we demonstrated that in a combined sample of SMI patients and HC, TG seropositivity was associated with increased circulating neuron-specific enolase (NSE), a marker of neuronal injury, and interleukin-18 (IL-18), an innate immune mediator indicative of inflammasome activation^50^. Additionally, TG antibody levels correlated with elevated circulating C-reactive protein (CRP) and an increased B cell-activating factor to A proliferation-inducing ligand (APRIL) ratio, suggesting heightened systemic inflammation and central nervous system pathology, respectively^57^. Collectively, these findings highlight the multifaceted mechanisms through which TG can contribute to structural brain damage, underscoring the critical need to manage chronic infections to mitigate long-term neurological consequences.

Although not the primary focus of the present study, we observed that women exhibited higher baseline cortisol levels than men within both the SMI patient group and the HC. This finding aligns with results from a large study conducted on an unselected population, which similarly reported elevated baseline cortisol levels in women^66^. Further, we showed an inverse relationship between age and cortisol concentrations in both SMI patients and HC. This phenomenon may be attributed to the attenuated awakening response associated with increasing age, indicating that the morning peak in cortisol secretion becomes less pronounced^67^. Notably, our findings suggest that this pattern is consistent across both SMI patients and HC, possibly suggesting a shared biological response to aging in relation to cortisol dynamics.

Our study has several limitations. First, although we utilized a well-defined study population and accounted for potential confounders, we cannot rule out the influence of unmeasured confounders that we could not adjust for. Additionally, due to the cross-sectional design, we are unable to establish causality in the observed associations between TG seropositivity, cortisol levels, and symptom scores. Moreover, the inverse relationship between TG seropositivity and psychotic symptom scores in SZ patients with a long DOI was unexpected, and we cannot exclude the possibility that this finding may be due to chance, despite previous reports showing non-significant trends in a similar direction^11^. Lastly, it is important to note that there was a difference in the timing of blood collection between the SMI patients and HC, with patients providing samples earlier in the day. However, since we conducted all analyses separately for the SMI and HC groups, we believe this discrepancy is unlikely to have confounded our results. Nonetheless, this limitation should be acknowledged, as future studies may benefit from standardizing the timing of sample collection to further minimize potential biases.

The potential clinical implications of our findings warrant consideration. If latent TG infection contributes to dysregulation of the HPA axis and subtle structural brain changes in patients with SMI, this could represent a modifiable factor influencing stress responsivity, illness progression, and treatment outcomes. Although we found an unexpected association between TG seropositivity and lower psychotic symptom scores in patients with longstanding SZ, it is possible that latent TG infection interacts with chronic antipsychotic exposure to alter disease trajectories or treatment responsivity over time. Furthermore, the observed associations with elevated cortisol levels may have implications for the somatic health of patients, given the established links between sustained hypercortisolemia, metabolic syndrome, and cardiovascular morbidity, which are already prevalent in individuals with SMI. On an individual level, awareness of chronic infectious exposures and their potential neuropsychiatric consequences could eventually inform risk stratification, monitoring strategies, or adjunctive therapeutic interventions. While the clinical utility of TG screening or targeted treatment in SMI remains speculative at present, our findings highlight the importance of considering infectious and immune-related factors when addressing the complex biopsychosocial needs of this vulnerable population.

In conclusion, TG seropositivity, reflecting latent toxoplasmosis, appears to be associated with dysfunction of the hypothalamus-pituitary-adrenal axis, as evidenced by elevated circulating baseline cortisol levels, particularly in SMI. This finding suggests a potential susceptibility to the adverse effects of TG in this patient cohort. Notably, latent toxoplasmosis in SZ patients with a prolonged DOI was associated with lower psychotic symptom scores, which may result from the chronic use of antipsychotic medication or a possible influence of the parasite on brain function and behaviour. Our findings raise important questions regarding the purported detrimental effects of TG on SMI, indicating that its impact may be more nuanced than previously assumed.

## Supplementary information

Suppl material

## Data Availability

Data supporting the findings of the present study are stored at NORMENT/Oslo University Hospital. Restrictions apply to the availability of data, and they are thereby not publicly available. Data can be made available under reasonable request and with permission of NORMENT/Oslo University Hospital, in accordance with the ethics agreements/research participants consent.
