# Supplementary material for: Toxoplasma gondii associated with psychotic symptom load and cortisol in severe mental illness
Source: Schizophrenia (Heidelb). 2025 May 26;11(1):80. doi: 10.1038/s41537-025-00630-0 (PMC12106729; doi:10.1038/s41537-025-00630-0)
Supplement: Supplementary file 1 — Suppl material [file 41537_2025_630_MOESM1_ESM.pdf]

Suppl. Material

Suppl. notes

ANCOVA assumptions for main models with statistically significant results

In the ANCOVA on PANSS total score among SMI patients, there was a deviation from the homogeneity of variances evaluated with Levene's test, p=0.032. The residuals of the overall model were approximately normally distributed determined by visual inspection. Observations with studentized residuals greater than 3 in absolute value were considered outliers. There were 8 outliers. These cases did not exhibit high leverage or high Cook's distance value and were not excluded. Excluding the outliers, the TG-PANSS association was still significant (p=0.008). Due to the deviation from the homogeneity of variances assumption, we ran sensitivity analysis applying a Mann-Whitney U test on PANSS total score. TG+ patients had still significantly lower PANSS total score than TG– patients (p=0.028).

In the ANCOVA on PANSS total score among SZ patients, there was homogeneity of variances evaluated with Levene's test, p=0.126. The residuals of the overall model were approximately normally distributed determined by visual inspection. Observations with studentized residuals greater than 3 in absolute value were considered outliers. There were 3 outliers. These cases did not exhibit high leverage or high Cook's distance value and were not excluded. Excluding the outliers, the TG-PANSS association was still significant (p=0.003).

In the ANCOVA on cortisol concentrations among SMI patients, there was homogeneity of variances evaluated with Levene's test, p=0.976. The residuals of the overall model were approximately normally distributed determined by visual inspection. Observations with studentized residuals greater than 3 in absolute value were considered outliers. There were 5 outliers. These cases did not exhibit high leverage or high Cook's distance value and were not excluded. Excluding the outliers, the TG-cortisol association was still significant (p=0.001).

Immune markers

We previously showed that TG seropositivity was associated with increased interleukin 18 (IL-18) and neuron-specific enolase (NSE) concentrations indicating inflammasome activation and neuronal injury, respectively [1], and that among TG+ individuals, TG IgG concentrations were positively associated with high-sensitivity C-reactive protein (hs-CRP) [2], all in combined samples of patients with

severe mental illness and healthy controls. We here aimed to investigate whether hs-CRP, IL-18 or NSE mediated the TG seropositivity-cortisol associations.

Among all participants, neither of the selected immune markers was correlated with cortisol, assessed with Spearman's correlations (p=0.144, p=0.619 and p=0.346 for CRP, IL-18 and NSE, respectively). Restricting the analysis to SMI patients, there were still no significant correlations (corresponding p-values 0.366, 0.095 and 0.223). Further, in SMI, applying three age- and sex-adjusted ANCOVAs where we also adjusted for CRP, IL-18 or NSE, the associations between TG seropositivity and cortisol remained significant (p=0.003 for all three associations), while as in the bivariate analyses, the selected immune markers were not associated with cortisol. These results suggest that the TG-cortisol associations may not be mediated by immunological mechanisms, at least related to CRP, IL-18 or NSE.

	MAGNETOM Sonata	Signa HDxt
**Field strength**	1.5T	3T
**N (patients/controls)**	421 (278/143)	397 (136/261)
**Sequence name**	MPRAGE	FSPGR
**Echo time (ms)**	3,93	MinFull
**Repetition time (ms)**	2730	7,8
**Inversion time (ms)**	1000	450
**Flip angle**	7 °	12 °
**Voxel size (mm3)**	1.33x0.94x1	1x1x1.2

Suppl. Table 1. An overview of the scanner systems used and their T1-weighted MRI sequences

Suppl. references

1 Andreou, D., Steen, N. E., Morch-Johnsen, L., Jorgensen, K. N., Wortinger, L. A., Barth, C. *et al.* Toxoplasma gondii infection associated with inflammasome activation and neuronal injury. *Sci Rep* **14**, 5327, doi:10.1038/s41598-024-55887-9 (2024).

2 Andreou, D., Steen, N. E., Jørgensen, K. N., Ueland, T., Wortinger, L. A., Mørch-Johnsen, L. *et al.* Increased Herpes simplex virus 1, Toxoplasma gondii and Cytomegalovirus antibody concentrations in severe mental illness. *Translational Psychiatry* **14**, 498, doi:10.1038/s41398-024-03198-y (2024).
